# Determining surgical surge capacity with a hybrid simulation exercise

**DOI:** 10.3389/fpubh.2023.1157653

**Published:** 2023-10-16

**Authors:** Magnus Blimark, Yohan Robinson, Catharina Jacobson, Hans Lönroth, Kenneth D. Boffard, Kristina Lennquist Montán, Ilja Laesser, Per Örtenwall

**Affiliations:** ^1^Centre for Disaster Medicine, University of Gothenburg, Gothenburg, Sweden; ^2^Centre for Defence Medicine, Swedish Armed Forces, Gothenburg, Sweden; ^3^Sahlgrenska University Hospital, Gothenburg, Sweden; ^4^Milpark Hospital, University of the Witwatersrand, Johannesburg, South Africa; ^5^Department of Global Public Health, Karolinska Institute, Solna, Sweden

**Keywords:** surge capacity, hospital disaster preparedness, civil defence, simulation training, mass casualty incident

## Abstract

**Background:**

To help test and improve surgical surge capacity, mass casualty incident (MCI) exercises generate valuable information. Both large scale table-top exercises (TTX) and full-scale exercises (FSX) have limitations if you want to test an organisation’s capability and structure. A hybrid exercise incorporating the advantages of TTX and FSX is a possible way forward, but is no standardised exercise method, yet. This study aims at evaluating the exercise results to determine the feasibility of a hybrid TTX/FSX exercise for an organization’s capability and structure.

**Methods:**

A hybrid MCI simulation using moulaged figurants and simulation cards was designed, where the emergency department of a level 1 trauma centre receives 103 casualties over 4 h. After registration and triage, all casualties are expected to be resuscitated in real time and are transferred for further treatment inside the hospital (radiology, operating theatres, intensive care unit (ICU)/postop and wards). When reaching operation theatre, ICU or ward, figurants are replaced by simulation cards. Observers ensured that those procedures performed were adequate and adhered to realistic times. Use of resources (materials, drugs etc.) were registered. Primary endpoint was average time spent in the emergency department, from time of arrival, to transfer out. Secondary endpoints were related to patient flow and avoidable fatalities.

**Results:**

The hospital managed to deal with the flow of patients without collapse of existing systems. Operating theatres as well as ICU and ward beds were available at the end of the exercise. Several details in the hospital response were observed that had not been noticed during previous TTX.

**Conclusion:**

FSX have a valuable role in training, equipping, exercising, and evaluating MCI management. Hybrid simulations combining both FSX and TTX may optimise resource utilisation and allow more frequent exercises with similar organisational benefit.

## Introduction

Mass casualty incident (MCI) management aims at improving operational readiness. MCI preparedness requires that simulation exercises are used to identify and eliminate issues before an actual emergency occurs. Exercise recommendations and corrective actions are essential to improving response systems and mechanisms to manage emergencies effectively (1, 2).

Swedish surgical surge capacity was studied through a survey distributed to key surgical and anaesthesia/ICU staff in all Swedish emergency hospitals in 2015 (3). Quantitative measures were the number of trauma teams, surgical theatres and ICU beds that could be mobilized in an MCI situation within 24 h. Within this time frame Sweden as a nation could offer 375 surgical teams, 469 surgical theatres and 559 ICU beds. It must be kept in mind that these numbers depend upon a functioning infrastructure, with full availability of hospital staff, pharmaceuticals, medical supplies, and blood products. In a disaster or MCI, these conditions cannot be taken for granted (4).

As a follow-up to our previous study the regional surgical surge capacity of a level 1 trauma centre was investigated, using a full-scale exercise, in real-time throughout, with more than 100 moulaged casualties. The aim of this study was to test the feasibility of a hybrid simulation model and to analyse patient flow to identify areas of improvement in the surgical surge response of a large Swedish university hospital to a mass casualty incident.

## Methods

### Study design

This is an evaluation of a joint full-scale civilian-military exercise with 103 simulated trauma cases in a university hospital setting in Sweden. Verbal informed consent was obtained from all participants. Their participation was voluntary, and they could withdraw their participation in the exercise at any time without any necessary explanation.

All methods were carried out in accordance with relevant guidelines and regulations. The Västra Götaland regional government and the Sahlgrenska University Hospital approved the experimental protocols for this exercise (no. RS 2018-01264).

Informed consent was obtained from all personnel that evaluation teams, camera-teams, press and visitors would be present during the whole exercise. Anyone unwilling to accept those conditions could decline to participate in the exercise. The hospital took measures to separate real patients from the exercise patients. There was no proximity, nor were real patients or their relatives subject to media attention.

The planning process started 7 months prior to the exercise and required multiple planning conferences. The Swedish Armed Forces Center for Defense Medicine (Gothenburg, Sweden) created the scenarios, provided casualty cards, recruited participants, and transported them by road and air to Sahlgrenska University Hospital (SU). SU provided the staff, hospital premises and medical equipment to deal with the casualties. Medical staff from other hospitals in Sweden functioned as observers during the exercise.

### Setting

Gothenburg is the second largest city in Sweden with a population of 595,598 residents (2018), while the metropolitan area has a population that exceeds one million residents. Sahlgrenska University Hospital (SU) is the largest hospital in Sweden, with 17,000 employees and 2,300 beds. The hospital has several locations. This exercise targeted the main Sahlgrenska University Hospital (SU/Sahlgrenska) located in the city centre, only. This building complex houses several key functions regarding emergency care (percutaneous cardiovascular catheterization intervention lab, trauma center, stroke, neurosurgical and cardiothoracic emergencies), that had to be fully operational despite the exercise.

### Participation and simulation cards

The casualty participants were recruited mostly from military personnel but also hired “professional” actors (Casualty Resources Ltd.^®^, United Kingdom) were used. They were all moulaged, dressed in surplus military clothing and trained to act according to their injuries on the morning of the exercise. Also, a MacSim casualty simulation card (5, 6) was attached to each one of them, (but hidden), depicting the injuries as well as their initial vital signs. The MacSim system consists of a card where the patient’s condition is illustrated by vital signs used by ATLS (airway, respiratory rate, blood pressure, pulse, GCS) along the sides of the card (7). Injuries detectable during a clinical examination of the patient are illustrated by symbols in the center of the card. The reverse side of the card is for observers only and list all the injuries of the patient along with RTS/ISS/NISS scores as well as timelines for certain procedures to be performed to avoid fatal outcome or permanent disability (6). MacSim has previously been used to test hospitals in Stockholm (Sweden) and Milan (Italy) (5, 8).

### The scenario

The scenario was the following: on Thursday morning April 12th, 2018, a truck loaded with explosives detonates at 09.55 a.m. within the Gothenburg Garrison on a public display day. The explosion causes 103 casualties (plus an unspecified number of fatalities) with injuries like those seen during the Madrid terrorist bombings in 2004 (9).

Multiple military vehicles (armored ambulances as well as mini-vans) and two Blackhawk medevac helicopters were available for transport of the casualties to Sahlgrenska university hospital (distance 8 km).

Casualty evacuation started immediately using vehicles of opportunity. After a while military ambulances and Medevac helicopters arrived and Medevac crews started triaging and treating patients on route to the hospital.

All simulated casualties were to be managed as in real life, i.e., triaged, registered and managed according to the ATLS principles at the emergency department (10). Triage was performed according to Triage Sieve (11). The color coding of Triage Sieve typically involves using four distinct colors to categorize patients based on the severity of their injuries or medical conditions:

1. Red: breathing patients that required airway assistance or have a respiratory rate below 10 or 30 or more. Even patients where capillary refill is over 2 s are categorized as red.
2. Yellow: patients that are unable to walk and have a respiratory rate between 10 and 29 and capillary refill below 2 s.
3. Green: patients that can walk.
4. Black: patients that are not breathing even after airway attention.

The use of medical supplies as well as blood and medications were documented. Observers controlled and ensured that real times were adhered to when different procedures were performed. Apart from the recording done by the observers, staff documented patient management in the separate disaster medical record according to the hospital’s contingency plan. Observers were able to adjust the vital signs according to the management of the patient as well as declaring them deceased. Following management in the emergency department the casualties were transferred to their next destination (radiology, OR, ICU, wards, morgue) in sequence, or were discharged.

During the exercise, all participating sectors of the hospital were monitored and assessed by external observers who recorded and summarized activities ranging from direct patient treatment to the activities in the hospital’s command groups.

### Debriefing results

A questionnaire was distributed to the involved hospital staff during the hot wash-up immediately following the exercise. These were to be filled out and returned at a later occasion.

### Primary endpoint

Primary endpoint was average time spent in the emergency department.

### Secondary endpoints

As secondary endpoint the patient flow was analysed. The following were documented for each patient:

- Medical supplies, blood, and medication used.
- Radiological imaging performed.
- Number of operation theatres in use.

Furthermore, the proportion of avoidable fatalities – as judged by observers, based on contents of the simulation card – was analysed.

### Analysis

The primary endpoint and secondary endpoints are presented as mean values with standard deviation, stratified for triage level (red/yellow/green).

## Results

### Preparatory considerations

The decision was made to divide the emergency department into two separate sections, one allocated to the exercise while the other half managed “real patient” flow. During the exercise ambulances were diverted to the other Sahlgrenska university hospital locations. The baseline hospital occupancy for the exercise was determined on a Thursday at 10.00 a.m. a couple of weeks prior to the exercise (and to coincide with the projected starting time of the exercise and included bed occupancy in ICU, wards, and on-going surgical procedures).

Alongside the planning conferences hospital staffs were trained in triage as well as how to interpret the MacSim cards.

The department of radiology had postponed all elective examinations for the day and only operated their emergency services. Participants transferred for CT scans were loaded into the scanner, however without any contrast injection or radiation exposure.

The exercise defined the entrance of the surgical theatre, the ICU, the surgical ward, the morgue, or the exit in case of discharge as the “end-point” for the injured participants. Thereafter, within the hospital the MacSim cards were attached to white-boards representing the different destinations, and ongoing care.

### Time to mass casualty incident alert

At 10.05 am (10 min after the incident) Sahlgrenska hospital was alerted. A large explosion at Gothenburg Garrison had been reported to the regional Incident Officer on call. The regional Incident Officer declared an MCI. The message was passed to the hospital resulting in alerting the hospital incident command, and activation of the MCI plan. Simultaneously the first casualties arrived by spontaneous evacuation to the ambulance entrance of the ED. During the following 4 h the hospital received in total 103 casualties arriving by road vehicles and helicopters.

### Triage and transport

Triage and registration teams were quickly dispatched to the hallway between the ambulance entrance and the ED corridor. The triage sieve determined which zone the casualties were transferred to: red (immediate care), yellow (quick care), green (can wait), or black (dead) zone. Patients in the blue category (expectant with little chances of survival) were transfer to the red zone during the exercise because that category was not part of the hospital’s contingency plan. Triage sort was used by resuscitation teams at the different zones so that the right priority was given. Following triage, the casualties were transferred to the appropriate sector. After a while, a shortage of trolleys became apparent and casualties unable to walk were dragged along the floor or were transported sitting in office chairs or other wheeled equipment.

### Aeromedical evacuation

The helipad of the hospital is located on top of a parking garage, 400 m walking distance from the emergency department. The internal transport time through elevators and underground corridors is about 10 min. The standard procedure for the civilian helicopter medical crew is to follow the patient to the emergency department and to make the handover there.

The military Medevac helicopters always carried more than one patient and prioritized a rapid turnover, to be able to rapidly bring in new casualties. Thus, the handover needed to take place on the helipad, a difficult task due to noise level from running turbines and lack of protection from weather conditions. A total of 24 patients were transported by air (23%).

The area of the ED allocated to the exercise was divided into following four zones:

### Red zone

**Figure 1:**
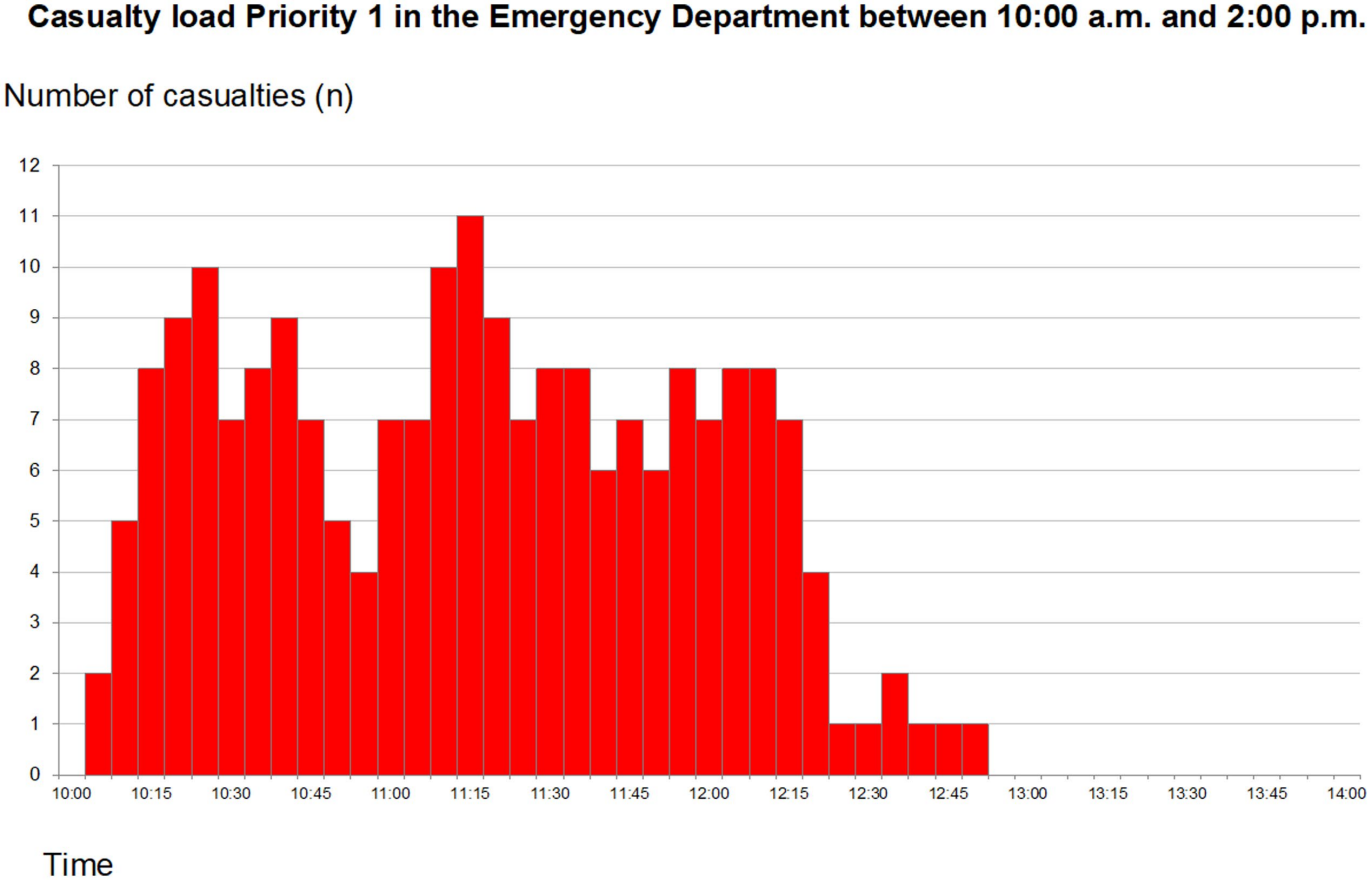
Total priority 1 (code red) patients arriving at the emergency department between 10:00 and 14:00. Reprint from Lennquist Montán et al. (7).

A total of 7 resuscitation teams were allocated to the red zone. The casualty load on this sector is presented in Figure 1. As can be seen the capacity was exceeded on several occasions. The mean time spent in the ED was 15 min and the mean waiting time for being surveyed was 1 min. Observers described the management as “high quality of care” and the figurants expressed that they “had felt seen by the staff.” Apart from the lack of trolleys sometimes necessitating medical care on the floor, limiting factors noted were limited access to ultrasound machines and insufficient Radiology staff able to operate radiological equipment. After a while, a lack of chest drains as well as surgical kits to insert these occurred. Attempts to have this shortage corrected resulted in lengthy discussions with the local hospital Incident Command, regarding the need for re-supply. With time, a shortage of portable oxygen, monitoring equipment and bags for ventilation occurred.

### Yellow zone

**Figure 2:**
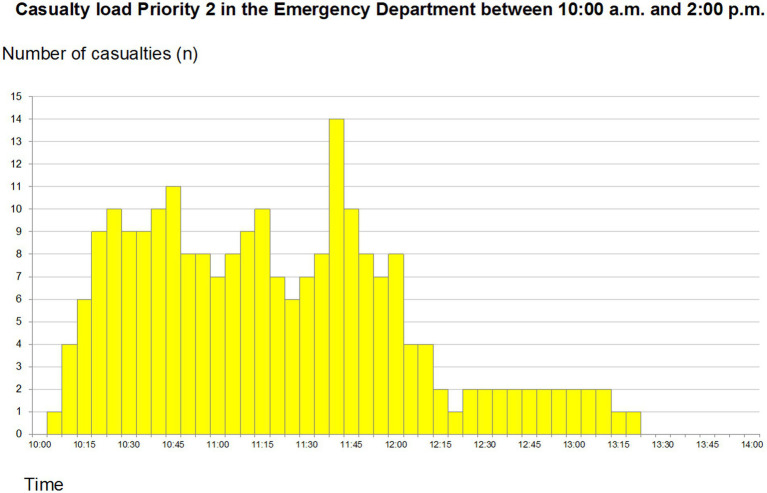
Total priority 2 (code yellow) patients arriving at the emergency department between 10:00 and 14:00. Reprint from Lennquist Montán et al. (7).

Three resuscitation teams were allocated to the yellow sector. The mean time spent in yellow sector was 36 min and the mean waiting time to be seen 6 min. This sector was under-staffed. When the patient load on the red sector increased, staff were re-allocated from the yellow to the red sector, occasionally leaving this sector without any physician present. The load on the yellow sector is presented in Figure 2.

### Green zone

**Figure 3:**
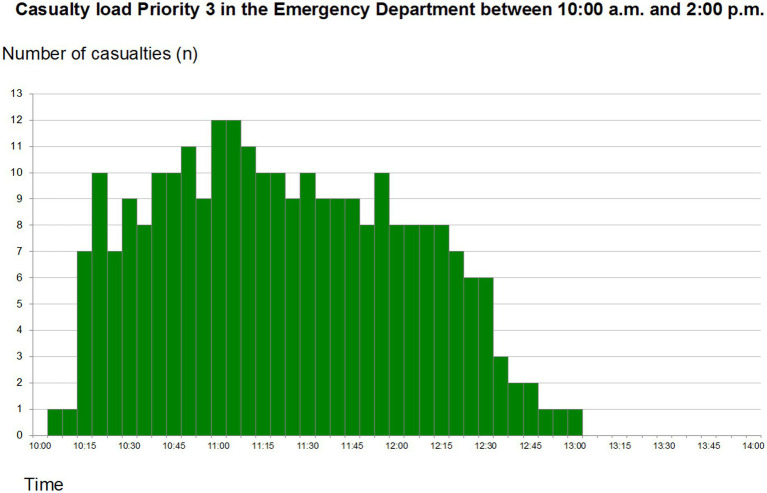
Total priority 3 (code green) patients arriving at the emergency department between 10:00 and 14:00. Reprint from Lennquist Montán et al. (7).

Only 1 resuscitation-team was allocated to the green sector, and they also “lost” their physician after a while due to the same reason as above. Some of the patients were re-triaged to a higher priority, but this sector had problems in transferring those to the appropriate sector. The casualty load is presented in Figure 3. The mean time spent in the ED was 71 min and mean waiting time to be seen 10 min 30 s.

### Blue zone

The blue (“Expectant”) category, usually reserved for patients whose chances of survival can be questioned even with maximum use of resources, was not part of the hospital’s contingency plan. However, during the exercise blue triage was implemented by the surgeon co-ordinating the activities in the red sector. Deceased patients would be transported to a dedicated area.

### Resources used

For airway management one patient required a tracheostomy, 21 patients had an endotracheal intubation, and 33 laryngeal masks were inserted. The limiting factors were not the airway equipment used, but (portable) monitors, oxygen and sometimes the skill necessary to perform the procedures.

There was a shortage of chest drains (34 inserted). However, the need for i.v. fluids (74 L) and analgesics (248 mg of morphine) as well as neck collars and other splints was fully met without problem.

### Radiology

Radiology is fully digitized and usually reports the result of an examination over the intranet of the hospital. However, according to the contingency plan they are supposed to deliver their reports on paper in case of an MCI.

Mobile teams as well as 8 CT-labs were available. The MacSim system provided slides from CT-scans of actual injuries for the radiologists to review.

All CT scans were performed according to the Swedish guidelines for Trauma CT (12). The average time for a CT scan was 7 min including load/unload time (reviewing time excluded).

Of the 103 casualties sixty had a “trauma-CT” performed. Only six other examinations were documented (3 chest and 3 extremity examinations). This “flooding” of the radiological department with casualties caused major problems. A waiting area outside the CT-labs had to be created. However, no preparations had been made as to how the casualties should be monitored and cared for while being in this area. In a few cases the trauma teams bringing the patient returned to the ED, leaving intubated and ventilated casualties without any monitoring. Such cases were declared as deceased by the observers.

Originally only one surgeon was stationed at the department of radiology with the task to decide on further patient care following the radiological examinations. This function became a bottle-neck and had to be reinforced by more surgeons (including a neurosurgeon) during the exercise.

### Operating theatres

SU/Sahlgrenska has approximately 40 operating theatres, 14 of these are large enough to be used for major trauma surgery (3). During the exercise these were physically co-located to one place and represented by whiteboards with the actual number of theatres depicted. The casualties were brought to the waiting area of this combined operating theatre and a handover was made to the surgeon/anesthesiologist in charge, re-triaging the patient. The participants were allowed to stand down leaving behind the casualty card, which was attached to a white board, corresponding to “waiting for surgery” or one of the available (staffed) operating theatres. Sketches of operative findings could be presented to the attending surgeons, followed by a discussion on how to handle the case. Since operating times for all procedures could be found on the back side of the MacSim card, the operating theatre was blocked for that period.

During the exercise 29 procedures were started (2 craniotomies, 5 thoracotomies, 16 laparotomies, 5 fasciotomies, and 1 wound debridement). There was always at least one OR available throughout the whole exercise.

One problem that surfaced after a while was shortage of sterile surgical instrument kits. Those responsible for packing and re-sterilizing of instruments had never been informed about the MCI. Since the staff in command of the operating theatres were very senior, they knew how kits could be “borrowed” from other surgical theatres in the hospital.

### ICU and postoperative ward

At the end of the exercise 28 patients had been admitted to the ICU/postop ward.

### Wards

The surgical wards received 31 patients. Two of these were transferred to the OR and 4 were discharged. At the end of the exercise 7 beds were still available. It was difficult for the co-ordinating nurse to keep track of the patient flow as well as the actual number of available beds. The presence of a surgeon in the wards to follow up on patient care is a requirement in the hospital’s contingency plan but functioned only intermittently during the exercise.

### Hospital incident command

Due to the size and multiple locations of Sahlgrenska university hospital the incident command was split into two incident commands – one jointly responsible for co-ordinating the work for all the hospital complexes within SU and one local incident command group responsible for work within SU/Sahlgrenska. These incident commands used different incident command rooms located in different parts of the building. To the observers it was not obvious how the tasks were divided between the two groups and which group had the mandate to decide on a specific topic. Communication between the groups seemed unstructured and time was spent discussing issues that were already regulated in the MCI plan.

### End of exercise

The exercise was stopped when the ED was cleared of casualties. All casualty cards and disaster medical records were collected, and the whiteboards photographed. Involved staff gathered for a debriefing giving a short summary of the activities of each involved department. A questionnaire was distributed requesting their further feedback of the exercise.

### Avoidable fatalities

Of the 103 casualties brought to the hospital 29 had a fatal outcome. Of these 11 cases were judged as unavoidable (4 due to injuries not compatible with survival and 7 due to late arrival to hospital). Eight fatalities were considered as possibly avoidable, however all of them except one having high ISS scores. The remaining 10 fatalities were more difficult to classify since they contain for example intubated patients left without monitoring in the radiological department.

### Feed-back from the trained staff

**Figure 4:**
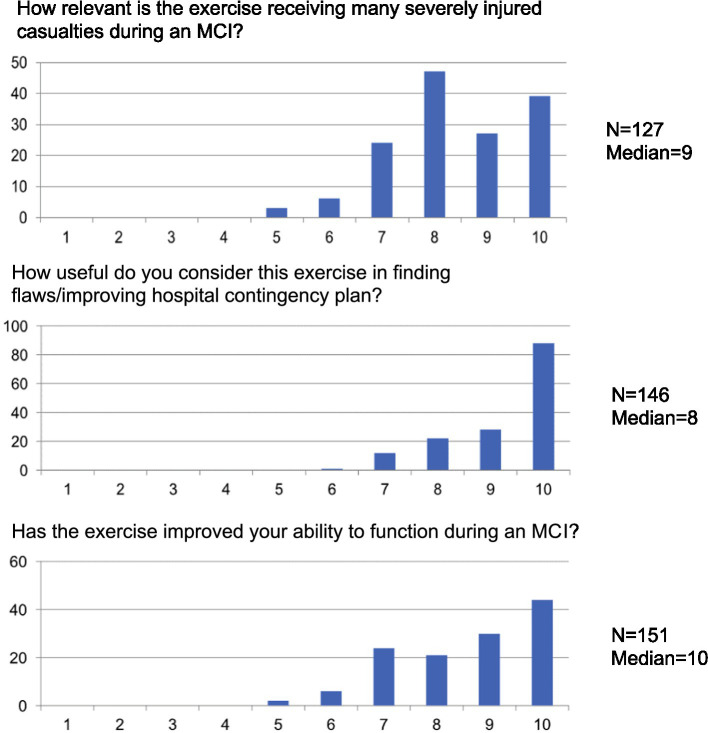
Summary of feedback from the trained staff (scaling for figures: 1, completely irrelevant; 10, very relevant).

The responses to the questionnaire during the hot wash-up immediately following the exercise are presented in Figure 4.

## Discussion

This study presents how a hybrid FSX/TTX design may improve the outcome of a MCI exercise. The hybrid exercise identified multiple structural and organisational issues, which would have been missed in a TTX, and staff reported high relevance regarding the MCI plan and own ability to function.

Surge capacity has been defined as the ability to obtain adequate staff, supplies and equipment, structures and systems to provide sufficient care to meet immediate needs of an influx of patients following a large-scale incident or disaster (13). Different countries are exposed to different kinds of threats reflecting their different approaches on how well contingency plans are organized, exercised, and implemented. Sweden has been spared from military conflicts, severe terrorist attacks and natural disasters for a long period, unlike many other countries of similar size and development (14). Even though legislation demands each emergency hospital to have a certain readiness as well as a contingency plan, the reality is that few hospitals have ever exercised this plan. One reason for this might be the costs associated with disaster preparedness (15). However, as the geopolitical situation has changed dramatically during the past decade, Sweden has been put in a context that has become more dangerous and unpredictable, raising the awareness for disaster preparedness and exercise needs (4).

An FSX with moulaged casualties is expensive (16). Many of the regular services of the hospital were reduced during this exercise involving in total almost 500 people, meaning loss of income from health care production. A TTX has lower costs since it can be conducted in rooms not directly involved in patient care (17). Such an exercise has the drawback of simplifying functions to be tested. For instance, communication as well as patient transport are much easier as compared to the real situation. The FSX led to the moving of the store for spare beds closer to the ED.

However, the key question is whether a hybrid FSX/TTX exposes bottlenecks in the disaster plan that would not be discovered during a TTX alone.

During the presented hybrid exercise, some important discoveries were made, that had not been found during previous TTX. The standard reception of patients delivered by helicopter does not work in an MCI. The crews prioritize a rapid turnover and cannot spend time transporting the patient inside the hospital. Thus, hospital medical staff – in sufficient numbers – need to meet at the helipad for a handover. This variation needs to be trained for and requires technical solutions for communicating in this noisy environment.

Within the ED a command function for matching and re-allocating staff based on the actual patient load was needed. The observed shortage of trolleys in the ED was first interpreted as a need for the hospital to purchase more trolleys. However, the return of existing trolleys back to the ED was not provided for in the MCI plan. If a patient was brought to the OR, ICU or ward, the trolley could be left there. Since the investigated hospital is a multilevel building, policing of the elevators was also needed.

The lack of crucial materials (i.e., chest drains, monitors) in the ED has been partially corrected after this exercise by stocking such items in carts in the ED, rotating them on a regular basis.

In the training of staff prior to the exercise, FAST ultrasound and plain radiographs were stressed to be the diagnostic procedures of choice in an MCI. However, more than 50% of the casualties were referred to a Trauma CT scan. Possibly this reflects that under stress most people act as they usually do in a similar clinical setting. Trauma CT has now become the standard examination for most “normal” trauma alerts in the hospital. Also, the referral became a problem since in some cases the CT was booked through the intranet, in some cases a paper referral was made and in some cases the team just brought the patient to the CT scanner. The disaster medical records are marked with a temporary ID number. It turned out that some of these temporary IDs had already been used by “real” patients and were then permanently linked in the software system to the “social security number” of an individual. Since radiology is fully digitized the equipment needs the registration of a valid ID before any examination can be performed. Some of the temporary IDs could not be used and as a result new temporary IDs had to be created, a time-consuming process markedly reducing the flow of patients through the radiology department.

The intention to deliberately overload the hospital to find where it would collapse failed. The assumption that more than 100 casualties would create a breakdown of care never happened. At the end of the exercise available operating theatres as well as ICU beds were still available.

A major problem for all involved sections of the hospital was communication. There is no radio network within the hospital (except for the porters). Instead, portable telephones were used as the major communication system. A telephone line only offers direct contact between two devices at a given time and during the exercise tended to be either busy or there was no answer to a call. It also turned out that some of the telephone numbers given for the exercise were incorrect, adding even more strain to the attempts of getting in contact with the different stakeholders. Instead, runners had to be used as back-up on multiple occasions. For internal information, the hospital’s intranet was used, projecting a red line on the opening page stating that the hospital had activated the MCI plan. Very few of the participating staff noted this and at the end of the exercise some of the nurses in the ED had no idea what type of incident they had been dealing with.

Another problem that became obvious was to pinpoint the exact location of patients at any given time. For instance, blood might be ordered from the ED, but when it arrived the patient had left for some other location in the hospital (radiology/OR/ICU) and no central function was able to keep track of patient movements. The same situation could occur if the written statement following a radiological examination was delayed.

During the planning phase, a suggestion was made to incorporate cleaning teams in the exercise. This was tested in the ED, where a team immediately following the transfer of a patient from a resuscitation cubicle entered and cleaned up the area. This was very appreciated by the medical staff and has been incorporated in the updated contingency plan, as well as being requested by radiology as well.

### Programs including full-scale exercises

Internationally, there are multiple programs which include both FSX and TTX in their educational program (18). One established program is the American National Disaster Life Support (NDLS) program that has been offering FSX and TTX since 2006 and incorporates standardized triage methodologies (19). Their training includes moulage victims, and simulation cards, which test the organization and community’s capabilities in the event of natural and man-made disasters. The participants respond to various mock scenarios. The use of disaster scenario-programmed human patient simulators responds in real-time to the events at hand. The first test of the triage involves sorting out victims to separate the less injured from the more injured, because the assumption is that the responders are responding under limited resources, including supplies, utilities, human resources, and laboratory capabilities with the aim to address the most seriously injured by identifying them.

Another program is the Interdisciplinary Emergency Service Cooperation Course (TAS) developed by the Norwegian Air Ambulance Foundation (20). The TAS-program was established in 1998. The TAS-concept is based on triage sieve and paediatric triage tape models but modified with slap-wrap reflective triage tags and paediatric triage stretchers. The TAS-triage concept is regularly trained and evaluated in full-scale simulated major incidents.

The European Master of Disaster Medicine (EMDM) program in Novara, Italy includes multiple TTX and a large civil-military prehospital FSX involving more than 130 moulaged figurants and a military field hospital (21). This FSX is not only educational but also a platform for research and development, where position tracking and responsive triage tags generate real-time research data (22).

Due to the limitations of FSX, alternative near-realistic simulations have been developed using virtual or augmented reality technology (23). Even though a near-realistic experience can be obtained, the simulation of human-human interaction is unsatisfactory so far. Therefore these solutions are mainly applicable for triage drills and operator training and not for testing a contingency plan of a healthcare system.

### Limitations of the study

SU/Sahlgrenska performed a TTX with identical types of casualties roughly 1 year prior to this exercise. Consequently, the hospital had time to prepare and could use the results from the TTX to critically examine their current MCI plan to correct identified errors.

The staff was allocated to the exercise before it took place and were standing by when the alert came. Since the exercise was run daytime on a Thursday, the hospital was fully staffed, and initial staff shortage was not anticipated to be a problem. However, the fixed number of staff engaged in the exercise made it impossible to scale up the teams, which might explain some of the shortcomings regarding monitoring of critically injured patients.

Resilience is critical to any contingency plan, and this was not examined during this exercise, which took place during a daytime shift, lasted 4 h in total, and ended when the last casualty had left the ED. It would have been valuable to continue the exercise with the casualty cards at the operation theatres, the intensive care units etc. to follow the activities in the hospital during the following days dealing with delayed surgeries, redo operations, staff rotations, re-supply, transfer of patients to other facilities as well as how to recover to normal operations of the hospital. On the other hand, since all white boards were photographed at the end of the exercise the set-up can be re-created for a follow-up tabletop exercise addressing these aspects.

## Conclusion

A mass casualty incident exercise using 103 moulaged casualties as well as simulation cards was successful in testing the surgical surge capacity of a level 1 trauma centre. The use of moulaged participants increased the stress perceived by involved staff and led to the discovery of several findings that had not been noticed during previous tabletop exercises. The exercise was highly rated by the trained hospital staff and will lead to changes of the contingency plan.

Future research needs to investigate observer learning effects from hybrid simulation exercises. As there were hospital staff members from other regional hospitals observing the FSX and TTX, their observations have affected their own hospitals’ contingency planning. Other areas for research are survivability measures for FSX evaluation, as for instance the unmonitored ventilation patients in this study. Finally, cost-effectiveness studies are needed to inform policy on costs and benefits of simulation exercises, to allow for effective planning and budgeting of FSX and hybrid simulation exercises.

## Data availability statement

The raw data supporting the conclusions of this article will be made available by the authors, without undue reservation.

## Ethics statement

Ethical review and approval was not required for the study on human participants in accordance with the local legislation and institutional requirements. Written informed consent from the participants was not required to participate in this study in accordance with the national legislation and the institutional requirements.

## Author contributions

CJ, KLM, and MB designed the study. HL and PÖ supervised the exercise execution. IL supervised the radiological activities. KLM and CJ developed the questionnaire. PÖ and KLM performed the analysis. KB and YR aided in interpreting the results and worked on the manuscript. All authors contributed to the article and approved the submitted version.

## Figures and Tables

**Table 1:** 

ATLS	Advanced trauma life support
CT	Computed tomography
ED	Emergency department
FAST	Focused assessment with sonography in trauma
FSX	Full scale exercise
GCS	Glasgow coma scale
GPS	Global positioning system
ICU	Intensive care unit
ISS	Injury severity score
Mascal	Mass casualties
MCI	Mass casualty incident
Moulage	Conventionally the term used to describe the makeup and simulated injuries of a participant such that the injuries seem “real.”
NISS	New injury severity score
Medevac	Medical evacuation
OR	Operation room
RTS	Revised trauma score
SU	Sahlgrenska University Hospital
TTX	Table-top exercise
